# Androgen receptor splice variants circumvent AR blockade by microtubule-targeting agents

**DOI:** 10.18632/oncotarget.4396

**Published:** 2015-06-22

**Authors:** Guanyi Zhang, Xichun Liu, Jianzhuo Li, Elisa Ledet, Xavier Alvarez, Yanfeng Qi, Xueqi Fu, Oliver Sartor, Yan Dong, Haitao Zhang

**Affiliations:** ^1^ College of Life Sciences, Jilin University, Changchun, P.R. China; ^2^ Department of Pathology, Tulane University School of Medicine, New Orleans, Louisiana, USA; ^3^ Department of Structural and Cellular Biology, Tulane University School of Medicine, New Orleans, Louisiana, USA; ^4^ Department of Medicine, Tulane University School of Medicine, New Orleans, Louisiana, USA; ^5^ Department of Urology, Tulane University School of Medicine, New Orleans, Louisiana, USA; ^6^ Tulane Cancer Center, Tulane University School of Medicine, New Orleans, Louisiana, USA; ^7^ Division of Comparative Pathology, Tulane National Primate Research Center, Covington, Louisiana, USA

**Keywords:** androgen receptor, splice variants, prostate cancer, taxane chemotherapy, microtubule

## Abstract

Docetaxel-based chemotherapy is established as a first-line treatment and standard of care for patients with metastatic castration-resistant prostate cancer. However, half of the patients do not respond to treatment and those do respond eventually become refractory. A better understanding of the resistance mechanisms to taxane chemotherapy is both urgent and clinical significant, as taxanes (docetaxel and cabazitaxel) are being used in various clinical settings. Sustained signaling through the androgen receptor (AR) has been established as a hallmark of CRPC. Recently, splicing variants of AR (AR-Vs) that lack the ligand-binding domain (LBD) have been identified. These variants are constitutively active and drive prostate cancer growth in a castration-resistant manner. In taxane-resistant cell lines, we found the expression of a major variant, AR-V7, was upregulated. Furthermore, ectopic expression of two clinically relevant AR-Vs (AR-V7 and AR^V567es^), but not the full-length AR (AR-FL), reduced the sensitivities to taxanes in LNCaP cells. Treatment with taxanes inhibited the transcriptional activity of AR-FL, but not those of AR-Vs. This could be explained, at least in part, due to the inability of taxanes to block the nuclear translocation of AR-Vs. Through a series of deletion constructs, the microtubule-binding activity was mapped to the LBD of AR. Finally, taxane-induced cytoplasm sequestration of AR-FL was alleviated when AR-Vs were present. These findings provide evidence that constitutively active AR-Vs maintain the AR signaling axis by evading the inhibitory effects of microtubule-targeting agents, suggesting that these AR-Vs play a role in resistance to taxane chemotherapy.

## INTRODUCTION

Prostate cancer is the most common non-skin cancer and the second leading cause of cancer mortality in men in the United States. Androgen deprivation therapy, which disrupts androgen receptor (AR) signaling by reducing androgen levels through surgical or chemical castration, or by administration of anti-androgens that compete with androgens for binding to AR [1], is the first-line treatment for metastatic and locally advanced prostate cancer. While this regimen is effective initially, progression to the presently incurable and lethal stage, termed castration-resistant prostate cancer (CRPC), invariably occurs. In 2004, docetaxel-based chemotherapy is established as a first-line treatment and standard of care for patients with metastatic CRPC [2]. However, about half of the patients do not respond to treatment and those do respond become refractory within one year. Several new treatments, including the new taxane cabazitaxel [3], the CYP17A1 inhibitor abiraterone [4], and the potent antiandrogen enzalutamide [5], have received FDA approval as second-line treatments for metastatic CRPC in recent years. However, the survival benefits are relatively small (< = 5 months) and patients eventually become refractory to treatments. Therefore, breakthroughs in the treatment of prostate cancer hinge upon better understandings of the mechanisms of therapeutic resistance of CRPC.

Paclitaxel, docetaxel, and cabazitaxel belong to the taxane family of chemotherapeutic agents. Taxanes bind to the microtubules and prevent their disassembly, thereby suppressing microtubule dynamics, leading to mitotic arrest and apoptosis [6]. This was believed to be the mechanism of action of taxanes in prostate cancer until recently when it was demonstrated by several groups that taxanes in fact inhibit the AR signaling pathway in prostate cancer. Taxanes have been shown to block the nuclear translocation of AR and inhibit the expression of AR-regulated genes [7, 8]. Additionally, Gan et al. showed that taxanes inhibit the transcriptional activity of AR by inducing FOXO1, a transcriptional repressor of AR [9]. It is well-established that CRPC cells remain addicted to AR signaling; therefore, the inhibitory effect on AR, rather than the antimitotic activity, could possibly be the predominant mechanism of action for taxanes in prostate cancer.

Sustained signaling through AR has been established as a hallmark of CRPC. Recently, alternative splicing variants of AR (AR-Vs) that lack the ligand-binding domain (LBD) have been identified [10–13]. These splice variants remain transcriptionally active in the absence of androgens and drive prostate cancer growth in a castration-resistant manner. In addition, these variants are reported to be prevalently upregulated in CRPC compared to hormone-naïve prostate cancer [10–13]. AR-Vs can regulate the expression of canonical androgen-responsive genes, as well as a unique set of target genes [12, 14]. In a significant portion of metastatic CRPC tissues, the variants proteins are expressed at a level comparable to that of the canonical, full-length AR (AR-FL) [15, 16]. Patients with high expression of two major AR-Vs, AR-V7 (also known as AR3) and AR^v567es^, have shorter cancer-specific survival than other CRPC patients [15]. In addition, recent studies have provided strong support for a critical role of these AR-Vs in resistance to hormonal therapies, including enzalutamide and abiraterone [17–20].

Recently, laboratory and clinical studies have suggested the existence of a cross-resistance mechanism between taxane-based chemotherapy and second-line hormonal therapies [21–25]. In this study, we set out to test the potential roles of AR-Vs in modulating the response to taxane-based chemotherapy.

## RESULTS

### Taxane-resistant prostate cancer cell lines express higher levels of AR-V7

**Figure 1:**
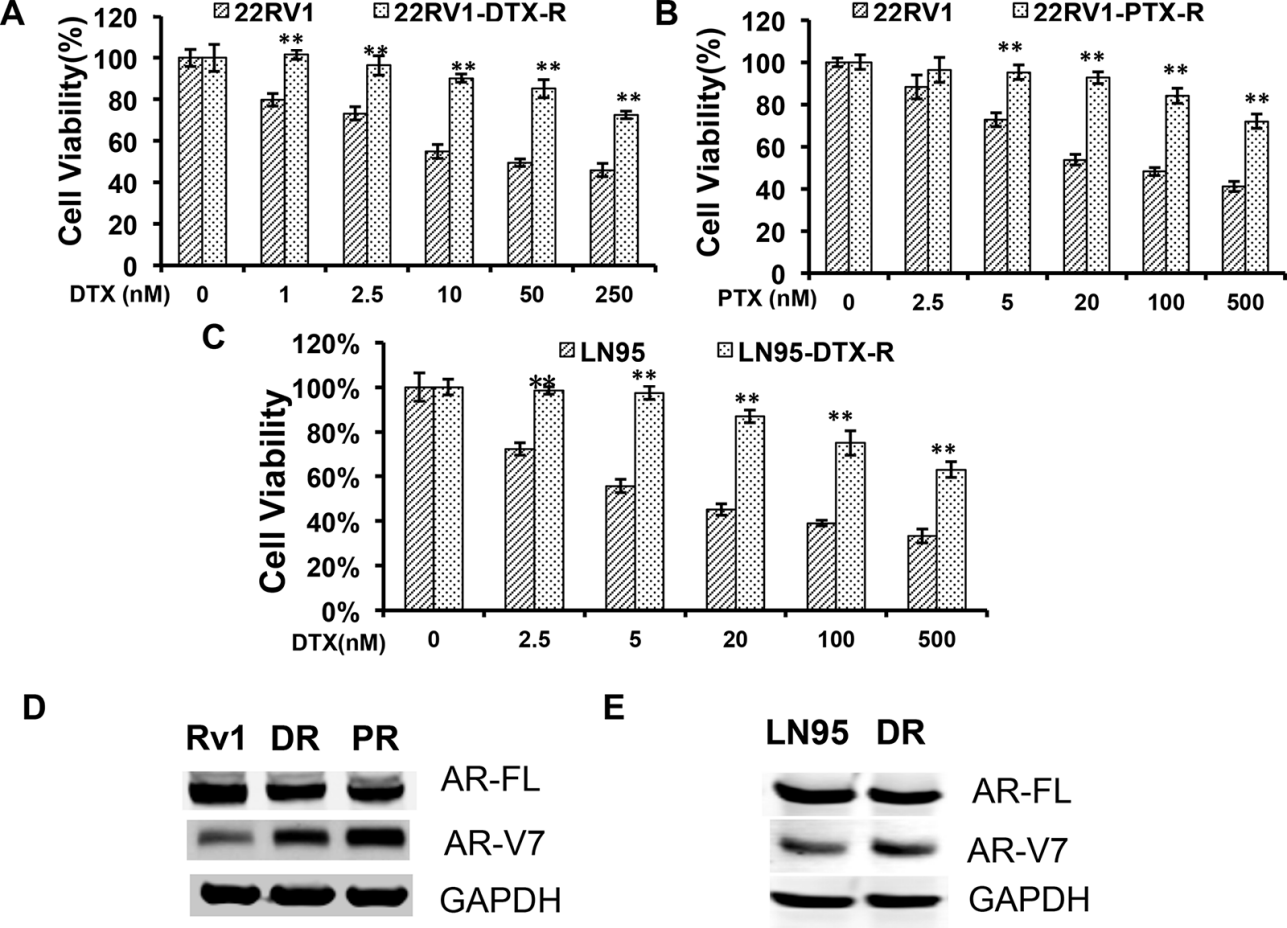
Upregulation of AR-V7 in taxane-resistant prostate cancer cells **A.** and **B.** 22Rv1 with acquired resistance to taxanes were established by culturing in escalating doses of docetaxel (DTX) or paclitaxel (PTX). MTT assays were performed in passage-matched 22Rv1 or 22Rv1 resistant cells to determine the responses to taxanes. **C.** The response of DTX-resistant LNCaP95 to docetaxel treatment. **D.** and **E.** Western blotting using an anti-N terminal antibody or an AR-V7-specific antibody in 22Rv1 (D) or LNCaP95 (E) resistant cells. Rv1/LN95, passage-matched parental line; DR, docetaxel-resistant; PR, paclitaxel-resistant. The *P* values were determined by the *Student's t*-tests, ** denotes *P* < 0.01. The results presented are mean ± SEM from three experiments.

We first established taxane-resistant 22Rv1 and LNCaP95 lines by culturing cells in escalating doses of paclitaxel and docetaxel over a period of 2 months. The response to taxanes were determined by the MTT assay (Fig. 1, A–C). Western blotting analyses showed that the expression of AR-FL was reduced, whereas the expression of AR-V7 was robustly induced, in the 22Rv1 resistant lines in comparison with the passage-matched parental line (Fig. 1D). A similar, albeit less pronounced, induction of AR-V7 was observed in the LNCaP95 docetaxel-resistant line (Fig. 1E). These results suggest that the constitutive active AR-V7 was selectively up-regulated in taxane-resistant prostate cancer cells.

### Expression of constitutively active AR-Vs impairs the cytotoxicity of taxanes

**Figure 2:**
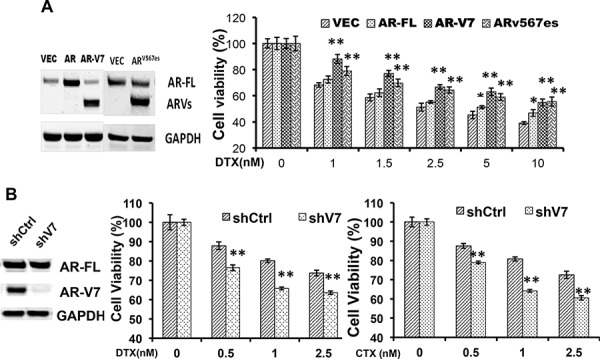
Expression of constitutively active AR-Vs negatively impact the cytotoxicities of taxanes **A.** LNCaP cells were transfected with vector, AR-FL, AR-V7, or AR^v567es^, and cell viability was determined by the MTT assay after 48 h of treatment with docetaxel. Western analysis was performed with an antibody recognizes the N-terminus of AR. The *P* values were determined by the *Student's t*-tests. *P < 0.05; **P < 0.01 vs vector. **B.** LNCaP95 cells were cultured in an androgen-depleted condition, and transfected with a control or an AR-V7-specific shRNA. **P < 0.01. CTX, cabazitaxel. The results presented are mean ± SEM.

To directly test the roles of constitutively active AR-Vs in resistance to taxanes, we transfected AR-V7 and AR^v567es^ into the AR-V-null LNCaP cells, and measured the responses to taxanes. As shown in Fig. 2A, cell viability after docetaxel treatment was markedly higher in cells expressing AR-V7 or AR^v567es^, but not in those overexpressing AR-FL, than in vector-transfected cells. Similar observations were made with paclitaxel and cabazitaxel (Supplementary Figure S1). In LNCaP95 cells, when the expression of AR-V7 was silenced by a V7-specific shRNA, cells became more sensitive to docetaxal and cabazitaxel (Fig. 2B). Taken together, these results suggest the expression of constitutively active AR-Vs negatively impacts the efficacies of taxanes in prostate cancer cells.

### Transcriptional activities of the constitutively active AR-Vs are refractory to the taxanes

**Figure 3:**
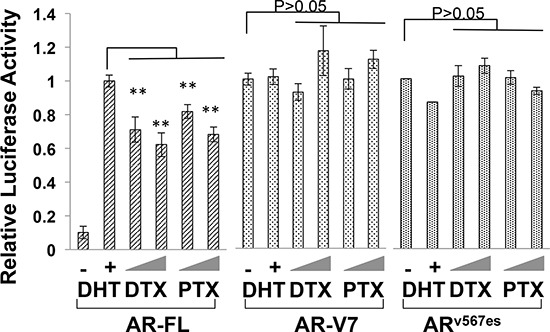
Transcriptional activities of constitutively active AR-Vs are refractory to taxane treatment COS-7 cells were transfected with the ARR3-luc reporter plasmid along with a plasmid encoding AR-FL, AR-V7, or AR^v567es^. The luciferase reporter assay was performed after 24 h treatment. The *P* values were determined by the *Student's t*-tests. **P < 0.01 vs untreated. Doses: DTX, 1 and 2.5 nM; PTX, 2.5 and 5 nM. The results presented are mean ± SEM from three experiments.

To understand the difference between AR-V7/AR^v567es^ and the AR-FL in cytoprotection against the taxanes, we investigated the influence of taxane treatment on the transactivation activities of these AR isoforms. COS-7, which does not express any AR proteins, was chosen in this experiment to avoid interference from the endogenous AR. As shown in Fig. 3, treatment with docetaxel or paclitaxel dose-dependently inhibited the ligand-dependent transcriptional activity of AR-FL, but neither drug was able to inhibit the constitutive activities of AR-V7 and AR^v567es^. This disparity can't be attributed to the down-regulation of AR-FL expression, as all AR proteins were not affected by the treatments (Supplementary Figure S2). These results suggest that the transcriptional activities of the AR variants are refractory to the inhibitory effects of taxanes.

### Nuclear imports of constitutively active AR-Vs are microtubule-independent

Next, we investigated the influence of the taxanes on nuclear translocation of AR-V7 and AR^v567es^, as these agents have been shown to block that of AR-FL [7, 8]. Enhanced green fluorescent protein (EGFP)-tagged AR-FL and AR-V7 were expressed in COS-7 cells and the localization of the fusion proteins was analyzed by fluorescence microscopy. Unlike EGFP-AR-FL, which required androgen stimulation for nuclear import, EGFP-AR-V7 spontaneously translocated to the nucleus (Supplementary Figure S3). When docetaxel and paclitaxel were added to the culture medium following androgen stimulation, accumulation of AR-FL in the cytoplasm was observed after 24 h of treatment (Supplementary Figure S3). However, treatment with the taxanes had no effect on the subcellular distribution of AR-V7.

**Figure 4:**
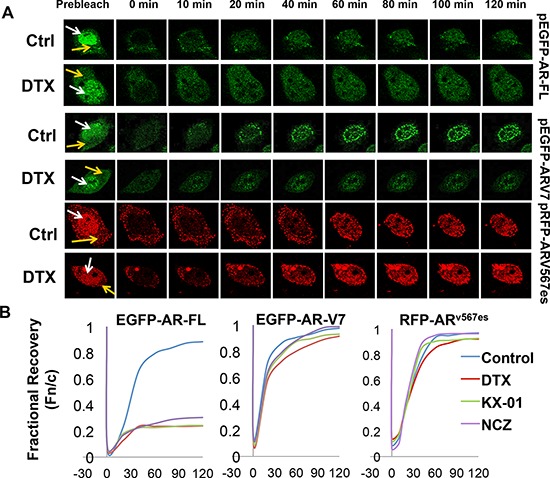
Nuclear imports of constitutively active AR-Vs are microtubule-independent FRAP assays were performed in COS-7 cells expressing different fluorescence-tagged AR proteins. Cells transfected with EGFP-AR-FL were cultured in the presence of androgen. Cells were treated with 20 nM docetaxel for 2 h before photobleaching. **A.** Confocal images taken at different intervals after photobleaching of the nuclei. White and yellow arrows indicate the nucleus and the cytoplasm, respectively. **B.** Recovery plot of the nuclear:cytoplamic fluorescence ratio (Fn/c) over time in cells treated with different microtubule inhibitors. Fn/c ratios are expressed as fractions of the pre-photobleach Fn/c. Nocodazole (NCZ) was used at 5 μg/ml and KX-01 was at 100 nM. FRAP images for NCZ and KX-01 are in Supplementary Figure S4.

To validate the results above, we performed fluorescence recovery after photobleaching (FRAP) assays in COS-7 cells expressing fluorescence-tagged AR proteins. Following treatment with docetaxel, selected nuclei were photobleached and the cells were imaged at regular intervals. Nuclear translocation is indicated by recovery of the nuclear to cytoplasmic fluorescence ratio (Fn/c). As indicated by the confocal images (Fig. 4A) and the fractional recovery plots (Fig. 4B), nuclear import of AR-FL was greatly deterred by docetaxel. In contrast, the nuclear translocations of AR-V7 and AR^v567es^ were not affected by docetaxel, evidenced by similar Fn/c recovery curves in control and treated cells (Fig. 4B). To substantiate these findings, we performed FRAP assays with additional microtubule inhibitors. KX-01 is a novel peptidomimetic inhibitor of Src family of kinases, but also inhibits tubulin polymerization [26], and nocodazole causes microtubule disassembly [27]. Once again, these drugs inhibited the nuclear import of AR-FL, but not that of AR-V7 or AR^v567es^ (Fig. 4B). Collectively, these results suggest the nuclear translocation of AR-V7 or AR^v567es^ are not mediated by the microtubules.

### AR associates with the microtubules through the LBD

**Figure 5:**
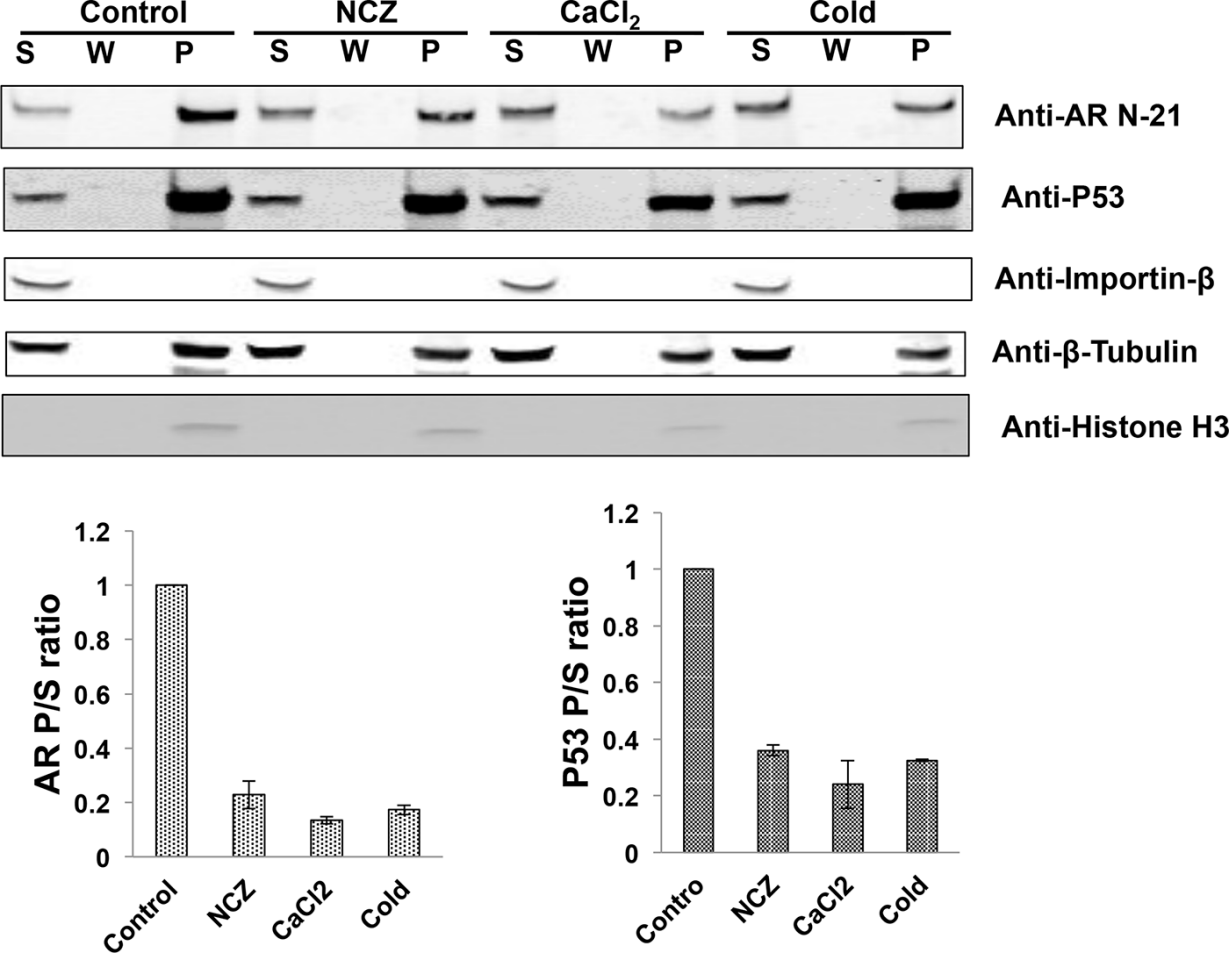
The full-length AR associates with the microtubules COS-7 cells were transfected with an expression vector for AR-FL and *in vivo* microtubule binding assay was performed with a commercial kit (Cytoskeleton, BK038). Nocodazole (NCZ), CaCl_2_, and low temperature (cold) were used to disrupt microtubule integrity. Assembled microtubules were precipitated by ultracentrifugation and the pellet was resuspended and analyzed by Western blot (Top). Importin β and p53 were used as negative and positive controls, respectively, and histone H3 was used to detect nuclear contamination. P, pellet; W, wash; S, supernatant. Bottom, the microtubule-binding activities for AR and p53 were quantitated by the P/S ratios. The results presented are mean ± SEM from three experiments.

Proteins that use the microtubule pathway for nuclear import are known to bind to the microtubules [28, 29]. To test whether AR binds to the microtubules, we conducted *in vivo* microtubule-binding assays in COS-7 cells ectopically expressing AR. Under the condition in which the microtubules were stabilized, the majority of AR-FL co-precipitated with the microtubules and was found in the pellet (Fig. 5). Importin β was used as a negative control as previously described [29], and p53, which is known to be a microtubule-binding protein [30], was used as the positive control. The microtubule-binding activity was quantitated by the pellet to supernatant (P/S) ratio [29]. In contrast, when nocodazole, CaCl_2_, or low temperature was employed to disrupt microtubule integrity, AR-FL shifted from the pellet to the supernatant, leading to marked decreases of the P/S ratios. These results suggest the AR-FL is a microtubule-associated protein.

**Figure 6:**
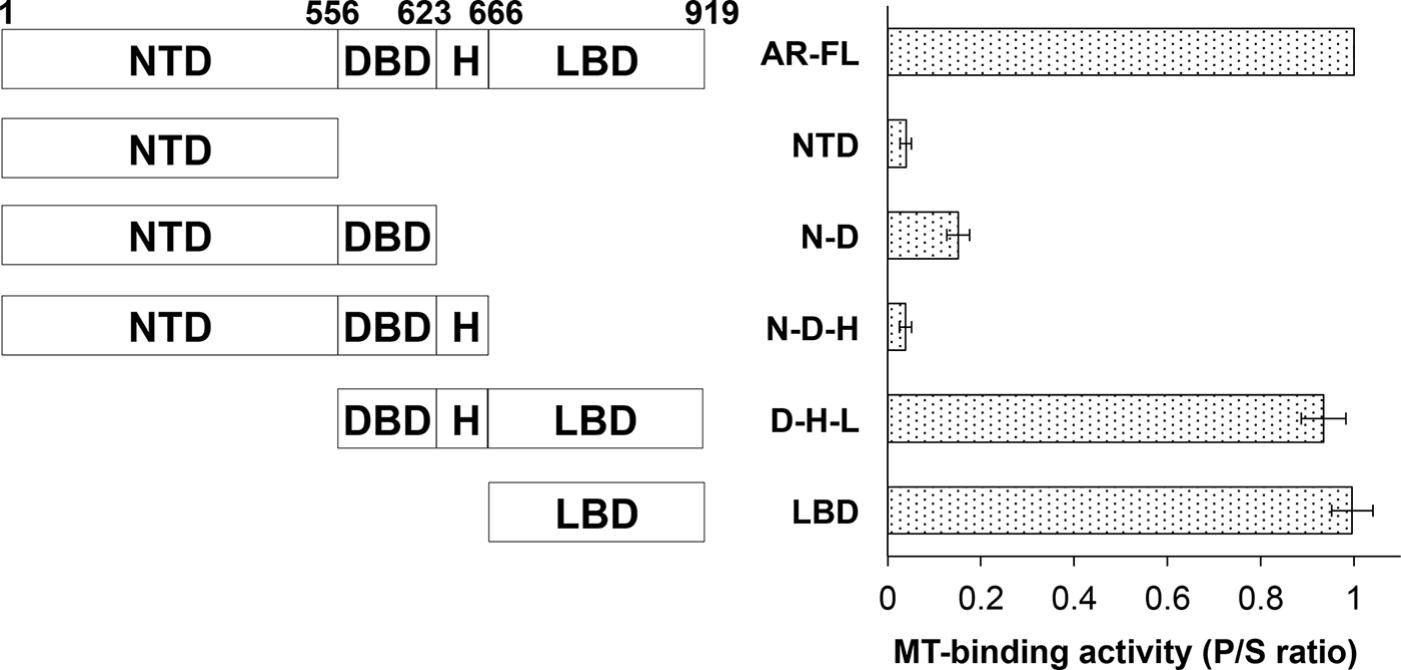
Microtubule-binding activity is mapped to the ligand-binding domain of AR Left panel, a series of deletion constructs encompassing different domains of AR were generated and expressed in COS-7 cells. Right panel, the microtubule-binding activities of these constructs were analyzed by the *in vivo* microtubule binding assay and the Western blots (Supplementary Figure S5) were quantitated to calculate the P/S ratios. The results presented are mean ± SEM from three experiments. MT, microtubule.

**Figure 7:**
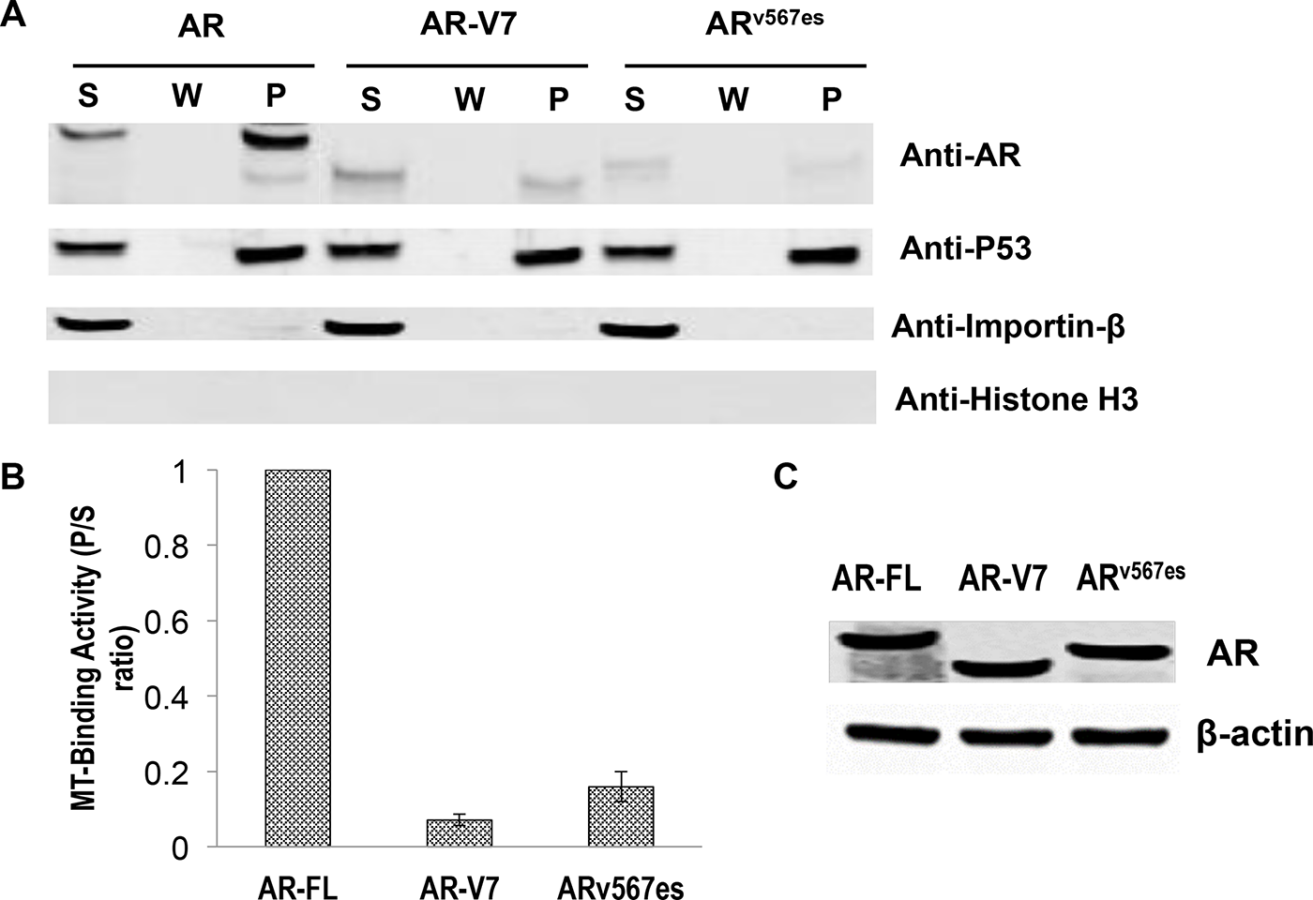
Poor microtubule-binding activities of the AR-Vs COS-7 cells were transfected with an expression vector for AR-FL, AR-V7, and AR^v567es^ and cultured in an androgen-deprived condition. **A.** *In vivo* MT-binding assays. **B.** quantitation of the results in A. The results presented are mean ± SEM from three experiments. **C.** Western blot showing that the proteins were expressed at similar levels after transfection.

To map the region responsible for microtubule-binding on AR, we generated a series of deletion constructs encompassing different domains of AR (Fig. 6, left panel). These constructs were analyzed by the microtubule binding assay. As shown in Supplementary Figure S4A and Fig. 6 (right panel), all constructs lacking the LBD have poor microtubule-binding activities. In contrast, those retaining the LBD have similar binding activities as that of AR-FL (Supplementary Figure S4B and Fig. 6). These results indicate that microtubule association is mediated by the LBD. Consistent with this finding, we found that the LBD-truncated AR-V7 and AR^v567es^ both bind poorly to the microtubules (Fig. 7).

### AR-Vs interfere with docetaxel-mediated AR-FL cytoplasmic retention

**Figure 8:**
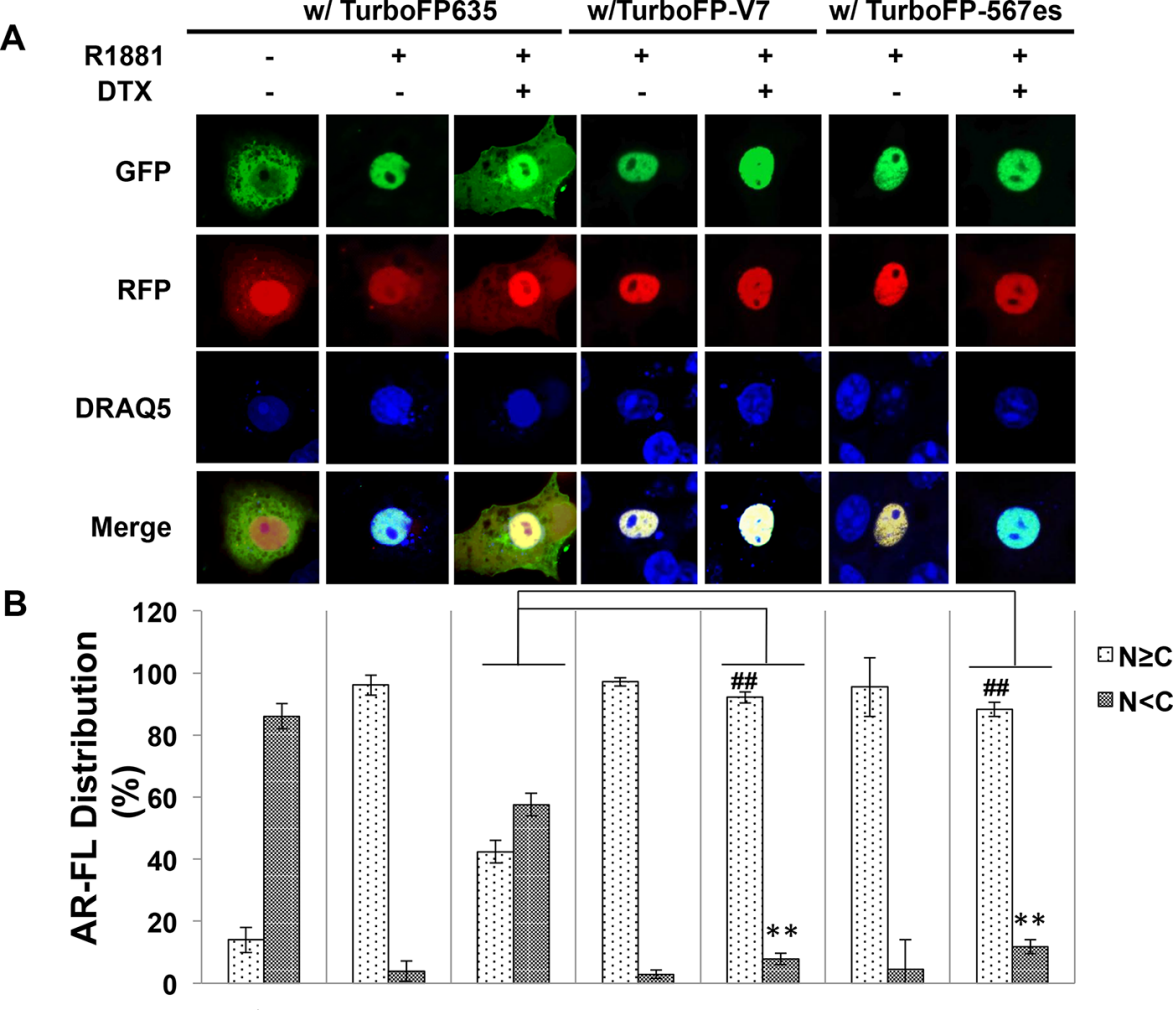

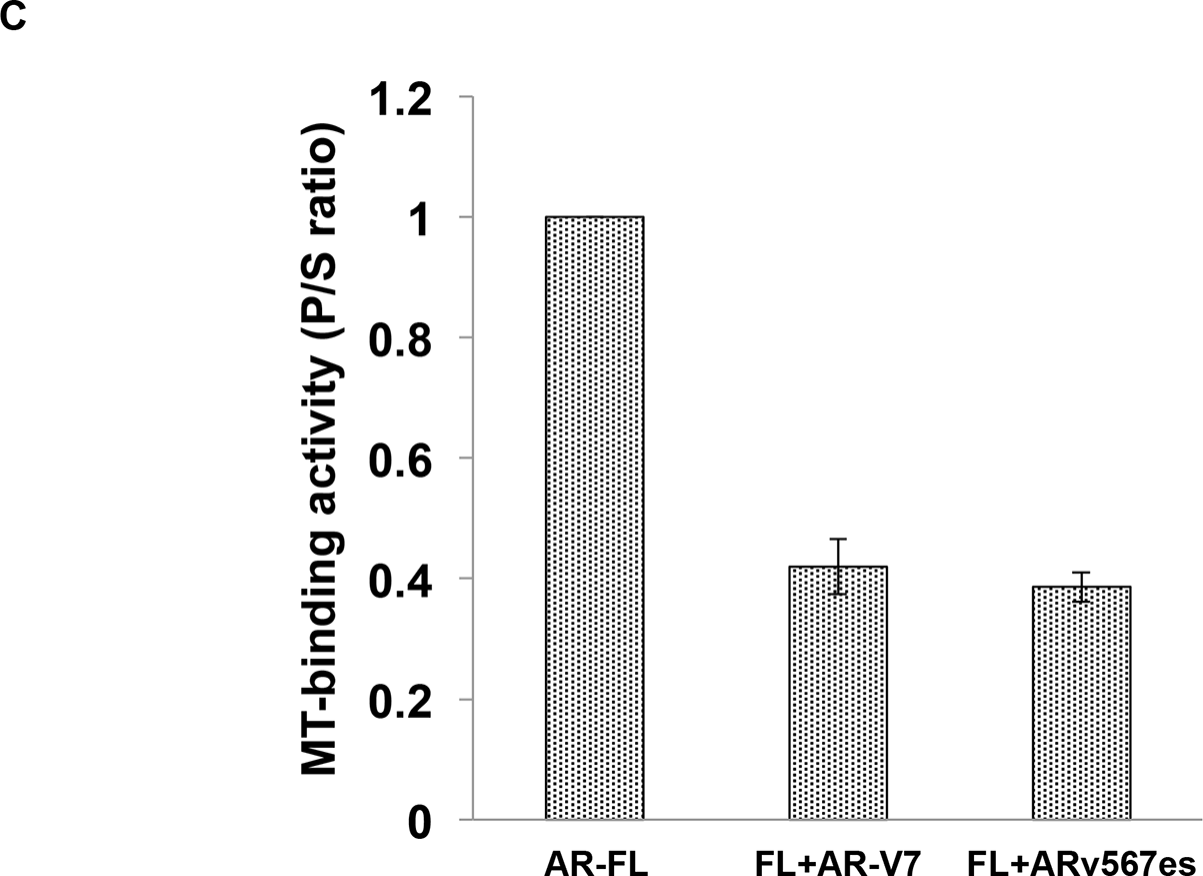
Cytoplasmic sequestration of AR-FL by docetaxel is attenuated by AR-V7 and AR^v567es^ **A.** Confocal fluorescence microscopy of EGFP-AR-FL subcellular localization when it was expressed with TurboFP or with a TurboFP-tagged AR-V in COS-7 cells. **B.** Based on distribution of the green fluorescence signal, cells were categorized into cytoplasmic (N < C), or nuclear and equally nuclear and cytoplasmic (N ≥ C).% of cells in each category were quantified. DRAQ5 was used to stain the nuclei. Cells cultured in an androgen-deprived condition were pre-treated with 10 nM docetaxel for 6 hr, followed by treatment with 1 nM R1881 for 4 hr. ** and ## *P* < 0.01. **C.** *In vivo* MT-binding assay in COS-7 cells expressing AR-FL alone, or with AR-V7 or AR^v567es^.

It has been previously shown that both AR-V7 and AR^v567es^ facilitate AR-FL nuclear translocation in the absence of androgen [13, 19]. To investigate whether AR-Vs mitigate the inhibitory effect of AR-FL nuclear translocation by docetaxel, we expressed EGFP-AR-FL with or without TurboFP635-tagged AR-V7 or AR^v567es^ in the AR-null COS-7 cells. When co-expressed with TurboFP635, EGFP-AR-FL was retained in the cytoplasm following docetaxel treatment (Fig. 8A). However, in the presence of AR-V7-TurboFP635 or AR^v567es^-TurboFP635, the inhibitory effect of docetaxel was significantly attenuated (Fig. 8A & 8B).

To further understand how AR-Vs circumvent docetaxel-mediated cytoplasmic sequestration of AR-FL, we conducted the microtubule-binding assay in COS-7 cells co-transfected with AR-FL and an AR-V. As shown in Fig. 8C, the binding of AR-FL to the microtubules was markedly reduced when it was co-expressed with AR-V7 or AR^v567es^. Taken together, these results suggest that the constitutively active AR-V7 or AR^v567es^ could divert AR away from the microtubules, and facilitate its nuclear translocation in a microtubule-independent manner.

### Nuclear import of AR-Vs is blocked by an importin β inhibitor

**Figure 9:**
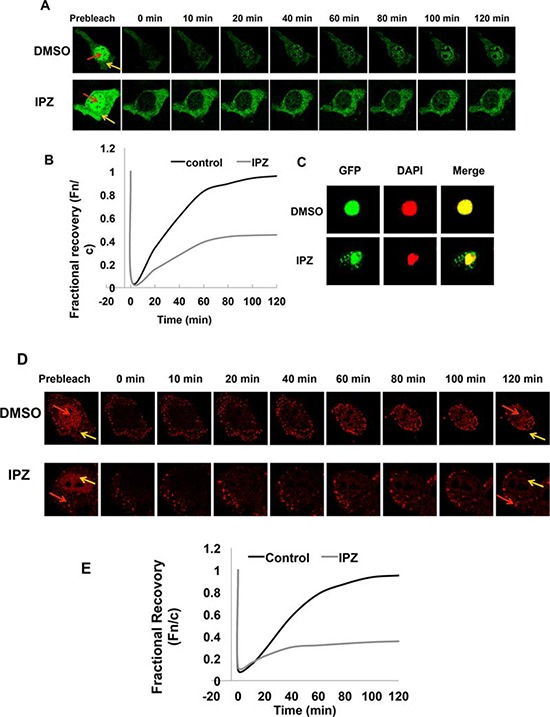
Nuclear translocation of AR-Vs is importin β-dependent **A.** FRAP assays were performed in COS-7 cells expressing EGFP-tagged AR-V7. Cells were treated with DMSO or 50 μM importazole (IPZ) for 2 h before photobleaching. Confocal images taken at different intervals after photobleaching of the nuclei. Red and yellow arrows indicate nucleus and cytoplasm, respectively. **B.** Fn/c recovery plot for EGFP-AR-V7. **C.** COS-7 cells transfected with pEGFP-AR-V7 were treated with DMSO or 10 μM importazole for 48 h. DAPI was used for staining the nuclei. **D.** & **E.** confocal images (D) and Fn/c recovery plot (E) of FRAP assays in COS-7 cells expressing TurboFP635-tagged AR^v567es^ and treated with IPZ. **D.** & **E.** confocal images (D) and Fn/c recovery plot (E) of FRAP assays in COS-7 cells expressing TurboFP635-tagged AR^v567es^ and treated with IPZ.

As an initial attempt to elucidate the nuclear translocation mechanisms of AR-V7 and AR^v567es^, we investigated the involvement of the importin α/β machinery. FRAP assay was conducted in COS-7 transfected with EGFP-AR-V7 and treated with importazole, a specific inhibitor of importin β [31]. As shown by Fig. 9A & 9B, treatment with importazole significantly reduced the recovery of AR-V7 in the nucleus. Consistently, AR-V7 was found to accumulate in the cytoplasm following importazole treatment (Fig. 9C). FRAP assay showed a similar inhibition by importazole on the nuclear recovery of TurboFP635-tagged AR^v567es^ (Fig. 9D & 9E), suggesting that both variants are imported to the nucleus by the importin α/β machinery.

## DISCUSSION

To date, docetaxel and cabazitaxel are the only chemotherapeutic agents that have been shown to offer survival benefits for patients with mCRPC. Even in today's rapidly evolving landscape of treatment options for mCRPC, taxane-based chemotherapy continues to be an important component of the treatment regimens. Recently, a randomized phase III trial supports the expansion of the indications of taxanes to earlier disease stages. The CHARRTED trial demonstrated that the addition of docetaxel to ADT in patients with high-volume, metastatic, hormonal-sensitive disease improves overall survival by 17 months (49.2 vs 32.2, *P* = 0.0013) than ADT alone [32]. With taxane chemotherapy projected to remain a mainstay in the treatment of prostate cancer, it is imperative to derive a better understanding of the mechanisms underlying the inherent and acquired taxane resistances, both of which are commonly observed in the clinic.

Resistance to taxanes could be multifactorial, involving general mechanisms of chemoresistance as well as mechanisms intrinsic to prostate cancer [33]. Existing literature focuses primarily on mechanisms common to many cancer types, including unfavorable tumor microenvironment, expression of drug efflux proteins, alterations in microtubule structure and/or function, expression of anti-apoptotic and cytoprotective proteins [34]. However, mechanisms that are specific to prostate cancer remain poorly understood. Recent clinical observations provided evidence for a cross-resistance of CRPC to hormonal therapy and taxane-based chemotherapy [21–25], suggesting a common culprit may underlie such a cross-resistance phenotype.

Our study represents a step forward in this direction. Herein, we present evidence that expression of constitutively active AR-Vs, but not over-expression of the canonical full-length receptor, protects prostate cancer cells from the cytotoxic effects of taxanes. We further show that taxane treatment selectively inhibits androgen-induced nuclear translocation and transactivation activity of AR-FL, while exerting no such inhibitory effects on the AR-Vs. These results reveal a fundamental difference in the nuclear translocation mechanisms of AR-FL and AR-Vs. AR-FL, as shown by this and other studies, utilizes a microtubule-facilitated pathway for nuclear translocation. This trafficking mechanism is shared by several nuclear proteins including glucocorticoid receptor (GR), p53, Rb, and parathyroid hormone-related protein (PTHrP) [29]. On the other hand, the nuclear import of AR-V7 and AR^v567es^ is not mediated by the microtubule pathway. The independence of the microtubule pathway enables the variants to evade taxane-induced cytoplasmic retention. Finally, we show that sequestration of AR-FL in the cytoplasm by taxanes is alleviated when AR-V7 or AR^v567es^ is present. This is likely caused by AR-V steering AR-FL away from the microtubules, as shown by reduced binding to the microtubules when AR-Vs are co-expressed. As an initial attempt to unveil the nuclear translocation mechanisms of the AR-Vs, we found that nuclear import of AR-V7 and ARv567es is possibly mediated by the importin α/β machinery. Elucidation of the upstream events will likely lead to opportunities to design novel strategies to target this variant.

The clinical relevance of AR-Vs has been demonstrated by a myriad of studies. Higher expression of AR-V7 in hormone-naïve prostate tumors predicts increased risk of biochemical recurrence following radical prostatectomy [11, 12], and patients with high levels of expression of AR-V7 or detectable expression of AR^v567es^ have a significantly shorter survival than other CRPC patients [15], indicating an association between AR-Vs expression and a more lethal form of prostate cancer. Studies have indicated that AR-Vs play important roles in resistance to androgen-directed therapies [17–19]. Particularly, a recent groundbreaking study by Antonarakis et al. showed that patients positive for AR-V7 expression in circulating tumor cells have significantly worse responses to enzalutamide or abiraterone than AR-V7-negative patients [20].

While the roles of AR-Vs are well recognized in resistance to hormonal therapies, evidence has just started to accumulate to support their involvement in resistance to taxane chemotherapy. Thadani-Mulero and colleagues are the first to show evidence supporting a role of AR-V7 in resistance to taxane chemotherapy [35]. In addition, the study by Martin et al. [36] showed that in cells harboring AR-Vs, targeting the AR N-terminal domain of with a small molecule inhibitor enhances the therapeutic response to docetaxel [36]. A clinical study by Steinestel et al. showed expression of AR-V7 in circulating cancer cells significantly correlates with prior treatment with docetaxel [37]. Very recently, a clinical study presented at the American Society of Clinical Oncology Genitourinary Cancers Symposium investigated the responses to taxane chemotherapy in mCRPC patients with different AR-V7 status in circulating tumor cells [38]. Although all the clinical outcomes are worse in patients in the AR-V7(+) arm, the differences are not statistically significant [38]. The insignificant differences could result from the small sample size or due to a “threshold effect” of AR-V7. In other words, the influence of AR-V7 on taxane response may be manifested only when it is expressed above a certain level. Hence, the association of AR-Vs and sensitivity to taxane chemotherapy warrants further investigation in a larger cohort.

The main disparity between our study and that of Thadani-Mulero et al. [35] is on whether AR^v567es^ is inhibited by the taxanes. In contrast to the data present herein, Thadani-Mulero and colleagues showed that AR^v567es^ associates with the microtubules and that the nuclear translocation of AR^v567es^ is inhibited by taxanes. In addition, the microtubule-binding activity is mapped to the DNA-binding and hinge domains of AR [35]. One possible explanation for these discrepancies is the use of different assays. Thadani-Mulero et al. performed *in vitro* assays in which cell lysates containing AR proteins tagged by GFP or hemagglutinin were incubated with purified tubulin in a cell-free system to allow microtubule polymerization and association. In contrast, we conducted *in vivo* microtubule-binding assays in which the microtubules and associated proteins were extracted from cells expressing untagged AR isoforms. Another major difference between the two studies is the dosage of taxanes. Docetaxel was applied at a concentration of 1 μM in the cell culture studies by Thadani-Mulero et al., in contrast to the clinically attainable [39] nanomolar concentrations used in our studies. We demonstrated that treatment with taxanes, at the low nanomolar concentrations, fail to inhibit the transcriptional activity or nuclear import of AR^v567es^.

The canonical AR nuclear localization signal (NLS) is located in the hinge domain, encoded by exons 3 and 4. Sequence analysis predicted that this NLS is truncated in AR-V7. However, the study by Chan et al. demonstrated that splicing of exon 3 with cryptic exon 3 in AR-V7 reconstitutes this bipartite NLS, which mediates the nuclear import of AR-V7 [40]. In addition, expression of a dominant negative mutant of Ran protein (RanQ69L) which causes premature dissociation of the importin/cargo complex, reduced nuclear localization of AR-V7 and AR^v567es^. These findings are consistent with our importazole data, suggesting that the nuclear import of the AR-Vs is mediated by the importin α/β pathway. They also found that unlike AR-FL, the nuclear localization of AR-V7 and AR^v567es^ is not affected by an inhibitor for heat shock protein 90. Together, this study and our data present herein suggest a fundamental difference between AR-FL and AR-Vs in the events upstream of importin α/β-mediated nuclear entry.

In summary, our study provides support for the involvement of AR-V7 and AR^v567es^ in attenuating the response to taxane-based chemotherapy. Mechanistically, we demonstrated that both variants translocate to the nucleus in a microtubule-independent manner. Additionally, these variants can reduce the microtubule-binding activity of AR-FL, thus circumventing its cytoplasm sequestration triggered by taxanes. These findings have important clinical implications. The expression status of these AR variants could potentially be used as a biomarker to aid treatment selection and sequencing. More importantly, targeting AR-Vs could be a fruitful direction to pursue to enhance the efficacy of taxane chemotherapy. To this end, several small molecule inhibitors at various stages of clinical development have shown promises against AR-Vs [41–43], opening doors for novel therapeutic strategies.

## MATERIALS AND METHODS

### Cell lines and reagents

LNCaP, 22Rv1, and COS-7 cells were obtained from American Type Culture Collection. With the exception of drug-resistant lines, cells used in this study were within 20 passages (∼3 months of non-continuous culturing). All cell lines were tested and authenticated by the method of short tandem repeat profiling. Docetaxel, cabazitaxel, and paclitaxel were purchased from Selleck Chemicals (Houston, TX). Nocodazole was from Sigma Aldrich, and KX-01 was provided by Kinex Pharmaceuticals. The following antibodies were used in Western blot analysis: anti-GAPDH, anti-AR (N-terminus-directed, PG-21; Millipore), anti-importin β1, anti-β-actin (Santa Cruz), anti-p53 (Calbiochem), anti-histone H3 (Cell Signaling), and anti-AR-V7 (Precision Antibody).

### Selection of taxane resistant cell lines

22Rv1 cells were initially treated with 10 nM paclitaxel for 72 hours and the surviving cells were re-seeded and allowed to recover for 1 week. Paclitaxel-resistant cells were developed over a period of 2 months by stepwise increasing concentrations of paclitaxel (5–50 nM). Age-matched parental cells which did not receive treatments were maintained in parallel. Docetaxel-resistant 22Rv1 and LNCaP95 lines were generated in a similar manner, but with different doses of docetaxel (5 nM initially, 2.5–20 nM for selection). The resistant cells were continuously maintained in the highest concentration of the taxane in which they selected.

### Western blotting

Cells were washed with ice-cold phosphate-buffered saline (PBS) and lysed with 2X Cell Lysis Buffer (Cell Signaling) containing a phosphatase inhibitor and the protease inhibitor cocktail (Sigma). After incubating the cells on ice for 30 min, lysates were collected by centrifugation at 10, 000 rpm for 10 minutes. Protein concentrations were determined by the BCA Protein Assay kit (Pierce). The samples were separated on 10% SDS-polyacrylamide gels and transferred onto polyvinylidene fluoride (PVDF) membranes. After blocking in TBS buffer (150 mM NaCl, 10 mM Tris, pH 7.4) containing 5% nonfat milk, the blots were incubated with a primary antibody overnight at 4°C and a fluorescent-labeled secondary antibody for 1 h at room temperature. The fluorescent signals were obtained by the Odyssey Infrared Imaging System (LI-COR Bioscience).

### Transient transfection and reporter gene assay

COS-7 cells were seeded in 10-cm dishes at a density to reach 80–90% confluency at time of transfection. Transient transfection was performed by using the Lipofectamine and Plus reagents following the manufacturer's instructions (Invitrogen). Cells were co-transfected with ARR3-luc luciferase reporter contruct and pRL-TK, along with a plasmid encoding for AR-FL, AR-V7 or AR^v567es^. After incubating with the transfection mixture for 4 h, cells were re-plated in RPMI 1640 containing 10% charcoal-stripped fetal bovine serum (cs-FBS). Cells were allowed to recover overnight before treated with DTX (1 and 2.5 nM) or PTX (2.5 or 5 nM) in the presence or absence of 10 nM DHT. Dual-luciferase assay was performed at 24 h post treatment using the Dual-luciferase Reporter Assay System (Promega). The renilla luciferase activity was used to normalize that of firefly luciferase.

### Confocal fluorescence microscopy

Subcellular localization of AR proteins was analyzed by confocal fluorescence microscopy. The pTurboFP635-AR-V7 and pTurboFP635-AR^v567es^ plasmids were generated by cloning the cDNA fragments for AR-V7 and AR^v567es^, respectively, into the pCMV-TurboFP635 vector. COS-7 cells were transfected with indicated plasmids and cultured in phenol red-free RPMI-1640 supplemented with 10% cs-FBS. At 40 hr after transfection, cells were pre-treated with or without 10 nM docetaxel for 6 hr, followed by treatment with or without 1 nM R1881 for 4 hr. COS-7 cells were subsequently fixed with 2% paraformaldehyde, and the nuclei were stained with 2.5 μM DRAQ5 (Cell Signaling). Confocal images were obtained by using a Leica TCS SP2 system with a 63X oil-immersion objective on a Z-stage, and an average of 6 fields with ∼10 cells per field were captured for each group. Data quantitation was performed as described [44].

### Fluorescence recovery after photobleaching (FRAP) assay

FRAP assay was performed using a Leica TCS SP2 microscope equipped with 20X, 40X and 63X oil immersion lenses (Nikon) in combination with a heated stage (Delta T Open Dish System, Bioptechs), as described by Roth et al. [45] with modifications. Briefly, three images were obtained before photobleaching using 10% of total laser power with excitation at 488 nm, scanning at a rate of 8 μs/pixel. Photobleaching was performed by scanning an area covering the entire nucleus 10 times at a rate of 12.5 μs/pixel, applying 100% of the laser power. After bleaching, the recovery of fluorescence was monitored by scanning the cells at 1 minute intervals for up to 2 hours, using detector and laser settings identical to those prior to photobleaching. Image analysis was carried out by using the NIH Image J Software to quantitate the nuclear (Fn) and cytoplasmic (Fc) fluorescence signals. The ratios of Fn to Fc (Fn/c) were calculated and the extent of recovery was determined by fractional recovery of Fn/c, which is the Fn/c at each time point divided by the prebleach Fn/c. The data were fitted exponentially to generate the fractional recovery plot.

### *In vivo* microtubule binding assay

The AR deletion constructs were generated by inserting PCR products of the corresponding cDNA regions into the pcDNA3.1(−) vector. The resulting plasmids were sequenced to confirm sequence accuracy and in-frame reading. COS-7 cells were transfected with indicated plasmids and cultured in RPMI 1640 medium supplemented with 10% cs-FBS. Microtubule-binding assay was performed by using the Microtubule/Tubulin *In Vivo* Assay Kit (Cytoskeleton Inc., Cat.# BK038) following the manufacturer's instructions. Briefly, 3 × 10^6^ cells were lysed in 4 mL pre-warmed (37°C) Lysis and Microtubule Stabilization 2 (LMS2) buffer (100 mM PIPES, pH 6.9, 5 mM MgCl_2_, 1mM EGTA, 30% (v/v) glycerol, 0.1% Nonidet P40, 0.1% Triton X-100, 0.1% Tween 20, 0.1% β-mercaptoethanol, 0.001% Antifoam, 100 μM GTP, 1 mM ATP, 1 × protease inhibitors cocktail) in a 10-cm cell culture dish. The lysates were collected and spun at 2,000 g for 10 min at 37°C to remove nuclei and unbroken cells. The supernatants were then subjected to ultracentrifugation at 100, 000 g for 30 min at 37°C to separate the microtubules from the soluble, unpolymerized tubulin. The pellet was washed with pre-warmed LMS2 buffer and centrifuged at 100, 000 g for 30 min at 37°C. For microtubule destabilization conditions, LMS2 buffer containing nocodazole (5 μg/ml) or CaCl_2_ (2 mM), or ice-cold LMS2 buffer were used in the above procedure. The pellets were resuspended in ice-cold 2 mM CaCl_2_ and incubated in room temperature for 15 min to depolymerize microtubules. The supernatant (S), wash solution (W), and resuspended pellet (P) were adjusted to equal volumes and analyzed by Western blotting.

### Statistical analysis

Statistical analysis was performed using Microsoft Excel. The *Student's* two-tailed *t*-test was used to determine the difference in means between two groups. *P* < 0.05 is considered significant. Data are presented as mean ± standard error of men (SEM).

## SUPPLEMENTARY FIGURES



## References

[1] Harris WP, Mostaghel EA, Nelson PS, Montgomery B (2009). Androgen deprivation therapy: progress in understanding mechanisms of resistance and optimizing androgen depletion. Nat Clin Pract Urol.

[2] Tannock IF, de WR, Berry WR, Horti J, Pluzanska A, Chi KN, Oudard S, Theodore C, James ND, Turesson I, Rosenthal MA, Eisenberger MA (2004). Docetaxel plus prednisone or mitoxantrone plus prednisone for advanced prostate cancer. N Engl J Med.

[3] De Bono JS, Oudard S, Ozguroglu M, Hansen S, Machiels JP, Kocak I, Gravis G, Bodrogi I, Mackenzie MJ, Shen L, Roessner M, Gupta S, Sartor AO (2010). Prednisone plus cabazitaxel or mitoxantrone for metastatic castration-resistant prostate cancer progressing after docetaxel treatment: a randomised open-label trial. Lancet.

[4] De Bono JS, Logothetis CJ, Molina A, Fizazi K, North S, Chu L, Chi KN, Jones RJ, Goodman OB, Saad F, Staffurth JN, Mainwaring P, Harland S, Flaig TW, Hutson TE, Cheng T, Patterson H, Hainsworth JD, Ryan CJ, Sternberg CN, Ellard SL, Flechon A, Saleh M, Scholz M, Efstathiou E, Zivi A, Bianchini D, Loriot Y, Chieffo N, Kheoh T, Haqq CM, Scher HI (2011). Abiraterone and increased survival in metastatic prostate cancer. N Engl J Med.

[5] Scher HI, Fizazi K, Saad F, Taplin M-E, Sternberg CN, Miller K, de Wit R, Mulders P, Chi KN, Shore ND, Armstrong AJ, Flaig TW, Fléchon A, Mainwaring P, Fleming M, Hainsworth JD, Hirmand M, Selby B, Seely L, de Bono JS (2012). Increased survival with enzalutamide in prostate cancer after chemotherapy. N Engl J Med.

[6] Jordan MA, Wilson L (2004). Microtubules as a target for anticancer drugs. Nat Rev Cancer.

[7] Zhu ML, Horbinski CM, Garzotto M, Qian DZ, Beer TM, Kyprianou N (2010). Tubulin-targeting chemotherapy impairs androgen receptor activity in prostate cancer. Cancer Res.

[8] Darshan MS, Loftus MS, Thadani-Mulero M, Levy BP, Escuin D, Zhou XK, Gjyrezi A, Chanel-Vos C, Shen R, Tagawa ST, Bander NH, Nanus DM, Giannakakou P (2011). Taxane-induced blockade to nuclear accumulation of the androgen receptor predicts clinical responses in metastatic prostate cancer. Cancer Res.

[9] Gan L, Chen S, Wang Y, Watahiki A, Bohrer L, Sun Z, Wang Y, Huang H (2009). Inhibition of the androgen receptor as a novel mechanism of taxol chemotherapy in prostate cancer. Cancer Res.

[10] Dehm SM, Schmidt LJ, Heemers HV, Vessella RL, Tindall DJ (2008). Splicing of a novel androgen receptor exon generates a constitutively active androgen receptor that mediates prostate cancer therapy resistance. Cancer Res.

[11] Hu R, Dunn TA, Wei S, Isharwal S, Veltri RW, Humphreys E, Han M, Partin AW, Vessella RL, Isaacs WB, Bova GS, Luo J (2009). Ligand-independent androgen receptor variants derived from splicing of cryptic exons signify hormone-refractory prostate cancer. Cancer Res.

[12] Guo Z, Yang X, Sun F, Jiang R, Linn DE, Chen H, Chen H, Kong X, Melamed J, Tepper CG, Kung HJ, Brodie AM, Edwards J, Qiu Y (2009). A novel androgen receptor splice variant is up-regulated during prostate cancer progression and promotes androgen depletion-resistant growth. Cancer Res.

[13] Sun S, Sprenger CC, Vessella RL, Haugk K, Soriano K, Mostaghel EA, Page ST, Coleman IM, Nguyen HM, Sun H, Nelson PS, Plymate SR (2010). Castration resistance in human prostate cancer is conferred by a frequently occurring androgen receptor splice variant. J Clin Invest.

[14] Hu R, Lu C, Mostaghel EA, Yegnasubramanian S, Gurel M, Tannahill C, Edwards J, Isaacs WB, Nelson PS, Bluemn E, Plymate SR, Luo J (2012). Distinct transcriptional programs mediated by the ligand-dependent full-length androgen receptor and its splice variants in castration-resistant prostate cancer. Cancer Res.

[15] Hornberg E, Ylitalo EB, Crnalic S, Antti H, Stattin P, Widmark A, Bergh A, Wikstrom P (2011). Expression of androgen receptor splice variants in prostate cancer bone metastases is associated with castration-resistance and short survival. PLoS One.

[16] Zhang X, Morrissey C, Sun S, Ketchandji M, Nelson PS, True LD, Vakar-Lopez F, Vessella RL, Plymate SR (2011). Androgen receptor variants cccur frequently in castration resistant prostate cancer metastases. PLoS ONE.

[17] Mostaghel EA, Marck BT, Plymate SR, Vessella RL, Balk S, Matsumoto AM, Nelson PS, Montgomery RB (2011). Resistance to CYPA1 inhibition with abiraterone in castration-resistant prostate cancer: induction of steroidogenesis and androgen receptor splice variants. Clin Cancer Res.

[18] Li Y, Chan SC, Brand LJ, Hwang TH, Silverstein KAT, Dehm SM (2013). Androgen receptor splice variants mediate enzalutamide resistance in castration-resistant prostate cancer Cell Lines. Cancer Res.

[19] Cao B, Qi Y, Zhang G, Xu D, Zhan Y, Alvarez X, Guo Z, Fu X, Plymate SR, Sartor O, Zhang H, Dong Y (2014). Androgen receptor splice variants activating the full-length receptor in mediating resistance to androgen-directed therapy. Oncotarget.

[20] Antonarakis ES, Lu C, Wang H, Luber B, Nakazawa M, Roeser JC, Chen Y, Mohammad TA, Chen Y, Fedor HL, Lotan TL, Zheng Q, De Marzo AM, Isaacs JT, Isaacs WB, Nadal R, Paller CJ, Denmeade SR, Carducci MA, Eisenberger MA, Luo J (2014). AR-V7 and resistance to enzalutamide and abiraterone in prostate cancer. N Engl J Med.

[21] Mezynski J, Pezaro C, Bianchini D, Zivi A, Sandhu S, Thompson E, Hunt J, Sheridan E, Baikady B, Sarvadikar A, Maier G, Reid AHM, Mulick Cassidy A, Olmos D, Attard G, de Bono J (2012). Antitumour activity of docetaxel following treatment with the CYP17A1 inhibitor abiraterone: clinical evidence for cross-resistance?. Ann Oncol Off J Eur Soc Med Oncol ESMO.

[22] Schweizer MT, Zhou XC, Wang H, Bassi S, Carducci MA, Eisenberger MA, Antonarakis ES (2014). The influence of prior abiraterone treatment on the clinical activity of docetaxel in men with metastatic castration-resistant prostate cancer. Eur Urol.

[23] Van Soest RJ, van Royen ME, de Morrée ES, Moll JM, Teubel W, Wiemer EAC, Mathijssen RHJ, de Wit R, van Weerden WM (2013). Cross-resistance between taxanes and new hormonal agents abiraterone and enzalutamide may affect drug sequence choices in metastatic castration-resistant prostate cancer. Eur J Cancer.

[24] Nadal R, Zhang Z, Rahman H, Schweizer MT, Denmeade SR, Paller CJ, Carducci MA, Eisenberger MA, Antonarakis ES (2014). Clinical activity of enzalutamide in docetaxel-naïve and docetaxel-pretreated patients with metastatic castration-resistant prostate cancer. The Prostate.

[25] Cheng HH, Gulati R, Azad A, Nadal R, Twardowski P, Vaishampayan UN, Agarwal N, Heath EI, Pal SK, Rehman HT, Leiter A, Batten JA, Montgomery RB, Galsky MD, Antonarakis ES, Chi KN, Yu EY (2015). Activity of enzalutamide in men with metastatic castration-resistant prostate cancer is affected by prior treatment with abiraterone and/or docetaxel. Prostate Cancer Prostatic Dis.

[26] Anbalagan M, Ali A, Jones RK, Marsden CG, Sheng M, Carrier L, Bu Y, Hangauer D, Rowan BG (2012). Peptidomimetic Src/pretubulin inhibitor KX-01 alone and in combination with paclitaxel suppresses growth, metastasis in human ER/PR/HER2-negative tumor xenografts. Mol Cancer Ther.

[27] Cassimeris LU, Wadsworth P, Salmon ED (1986). Dynamics of microtubule depolymerization in monocytes. J Cell Biol.

[28] Roth DM, Moseley GW, Glover D, Pouton CW, Jans DA (2007). A microtubule-facilitated nuclear import pathway for cancer regulatory proteins. Traffic.

[29] Roth DM, Moseley GW, Pouton CW, Jans DA (2011). Mechanism of microtubule-facilitated “fast track” nuclear import. J Biol Chem.

[30] Giannakakou P, Sackett DL, Ward Y, Webster KR, Blagosklonny MV, Fojo T (2000). p53 is associated with cellular microtubules and is transported to the nucleus by dynein. Nat Cell Biol.

[31] Soderholm JF, Bird SL, Kalab P, Sampathkumar Y, Hasegawa K, Uehara-Bingen M, Weis K, Heald R (2011). Importazole, a small molecule inhibitor of the transport receptor importin-β. ACS Chem Biol.

[32] Sweeney C, Chen Y-H, Carducci MA, Liu G, Jarrard DF, Eisenberger MA, Wong Y-N, Hahn NM, Kohli M, Vogelzang NJ, Cooney MM, Dreicer R, Picus J, Shevrin DH, Hussain M, Garcia JA, DiPaola RS (2014). Impact on overall survival (OS) with chemohormonal therapy versus hormonal therapy for hormone-sensitive newly metastatic prostate cancer (mPrCa): An ECOG-led phase III randomized trial. J Clin Oncol.

[33] Seruga B, Ocana A, Tannock IF (2011). Drug resistance in metastatic castration-resistant prostate cancer. Nat Rev Clin Oncol.

[34] Antonarakis ES, Armstrong AJ (2011). Evolving standards in the treatment of docetaxel-refractory castration-resistant prostate cancer. Prostate Cancer Prostatic Dis.

[35] Thadani-Mulero M, Portella L, Sun S, Sung M, Matov A, Vessella RL, Corey E, Nanus DM, Plymate SR, Giannakakou P (2014). Androgen receptor splice variants determine taxane sensitivity in prostate cancer. Cancer Res.

[36] Martin SK, Banuelos CA, Sadar MD, Kyprianou N (2015). N-terminal targeting of androgen receptor variant enhances response of castration resistant prostate cancer to taxane chemotherapy. Mol Oncol.

[37] Steinestel J, Luedeke M, Arndt A, Schnoeller TJ, Lennerz JK, Wurm C, Maier C, Cronauer MV, Steinestel K, Schrader AJ Detecting predictive androgen receptor modifications in circulating prostate cancer cells. Oncotarget.

[38] Antonarakis ES, Lu C, Chen Y, Luber B, Wang H, Nakazawa M, Marzo AMD, Isaacs WB, Nadal R, Paller CJ, Denmeade SR, Carducci MA, Eisenberger MA, Luo J (2015). AR splice variant 7 (AR-V7) and response to taxanes in men with metastatic castration-resistant prostate cancer (mCRPC). J Clin Oncol.

[39] Clarke SJ, Rivory LP (1999). Clinical pharmacokinetics of docetaxel. Clin Pharmacokinet.

[40] Chan SC, Li Y, Dehm SM (2012). Androgen receptor splice variants activate androgen receptor target genes and support aberrant prostate cancer cell growth independent of canonical androgen receptor nuclear localization signal. J Biol Chem.

[41] Andersen RJ, Mawji NR, Wang J, Wang G, Haile S, Myung JK, Watt K, Tam T, Yang YC, Banuelos CA, Williams DE, McEwan IJ, Wang Y, Sadar MD (2010). Regression of castrate-recurrent prostate cancer by a small-molecule inhibitor of the amino-terminus domain of the androgen receptor. Cancer Cell.

[42] Liu C, Lou W, Zhu Y, Nadiminty N, Schwartz CT, Evans CP, Gao AC (2014). Niclosamide inhibits androgen receptor variants expression and overcomes enzalutamide resistance in castration-resistant prostate cancer. Clin Cancer Res.

[43] Purushottamachar P, Godbole AM, Gediya LK, Martin MS, Vasaitis TS, Kwegyir-Afful AK, Ramalingam S, Ates-Alagoz Z, Njar VCO (2013). Systematic structure modifications of multi-target prostate cancer drug candidate galeterone to produce novel androgen receptor down-regulating agents as an approach to treatment of advanced prostate cancer. J Med Chem.

[44] Li J, Cao B, Liu X, Fu X, Xiong Z, Chen L, Sartor O, Dong Y, Zhang H (2011). Berberine suppresses androgen receptor signaling in prostate cancer. Mol Cancer Ther.

[45] Roth DM, Harper I, Pouton CW, Jans DA (2009). Modulation of nucleocytoplasmic trafficking by retention in cytoplasm or nucleus. J Cell Biochem.

