# Supplementary material for: Androgen receptor splice variants circumvent AR blockade by microtubule-targeting agents
Source: Oncotarget. 2015 Jun 22;6(27):23358–71. doi: 10.18632/oncotarget.4396 (PMC4695123; doi:10.18632/oncotarget.4396)
Supplement: Supplementary file 1 [file oncotarget-06-23358-s001.pdf]

SUPPLEMENTARY FIGURES

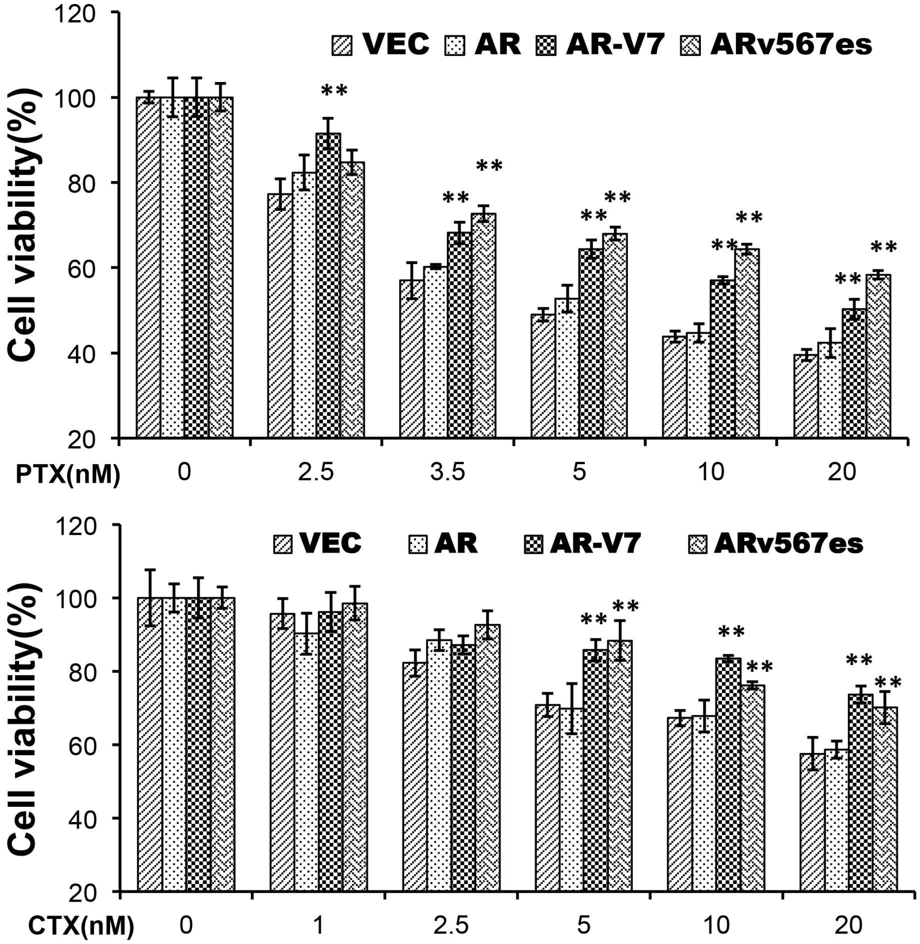

Supplementary Figure S1: Expression of constitutively active AR-Vs reduces the cytotoxicities of taxanes. LNCaP cells were transfected with vector, AR-FL, AR-V7, or AR^{v567es} as described in Figure 2 and cell viability was determined by the MTT assay after 48 h of treatment. **A.**, paclitaxel. **B.**, cabazitaxel. $^{**}P < 0.01$ vs vector-transfected.

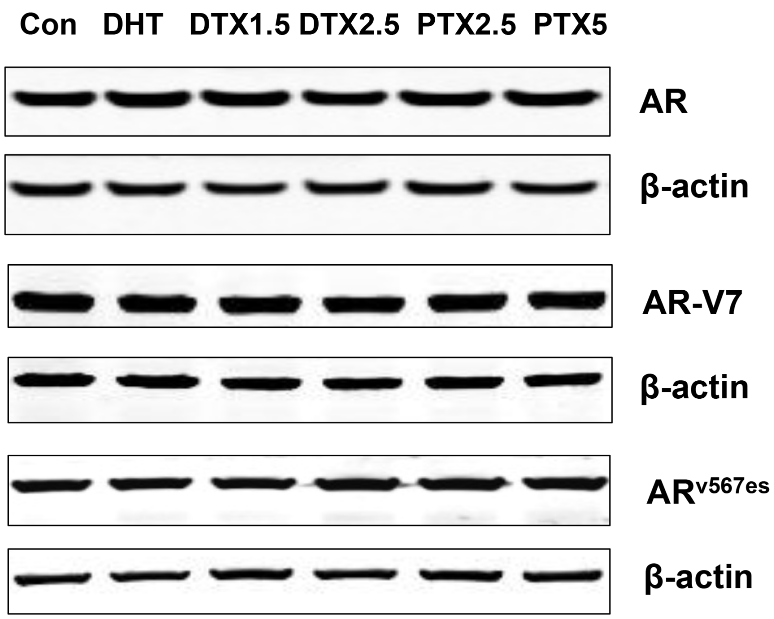

Supplementary Figure S2: Treatment with the taxanes does not affect the levels of transfected AR proteins in COS-7. Aliquots of the cell lysates used in the luciferase assays in Figure 3 were analyzed by Western Blotting to determine if the taxanes differentially affect the expressions of AR-FL and AR-Vs.

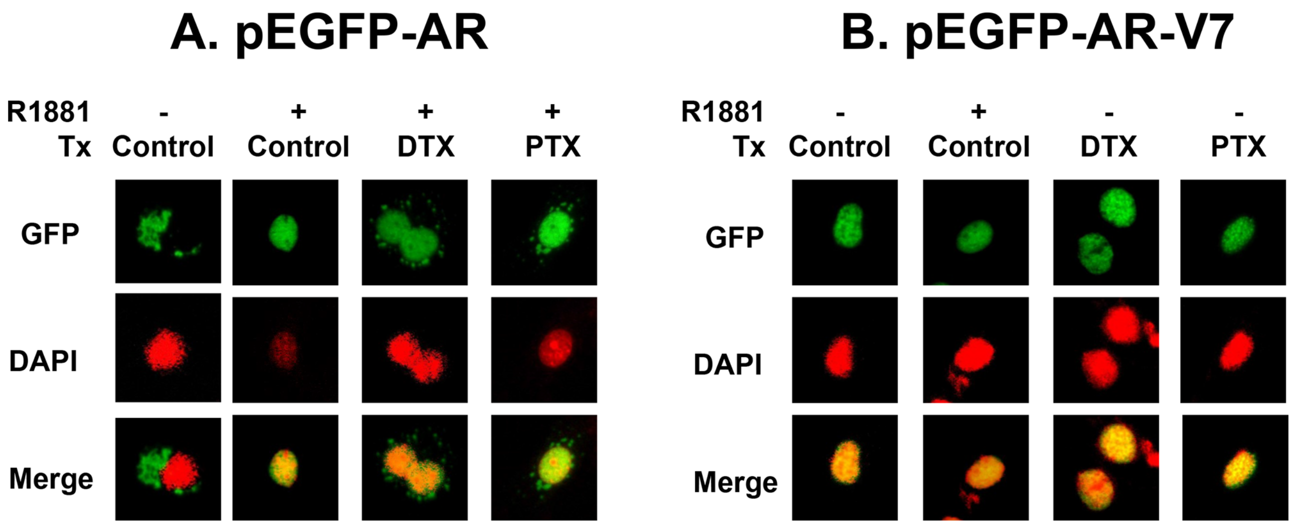

Supplementary Figure S3: Taxanes inhibit ligand-induced nuclear translocation of AR-FL, but have no effect on the spontaneous translocation of AR-V7. A., COS-7 cells transfected with pEGFP-AR-FL were initially cultured in an androgen-deprived condition. Treatment with docetaxel (10 nM) and paclitaxel (20 nM) started 2 h after the addition of R1881 (1 nM) to the culture medium, and images were taken after 24 h of treatment. DAPI was used for staining the nuclei. **B.**, COS-7 transfected with pEGFP-AR-V7 were cultured in an androgen-depleted condition and treated with docetaxel or paclitaxel for 24 h.

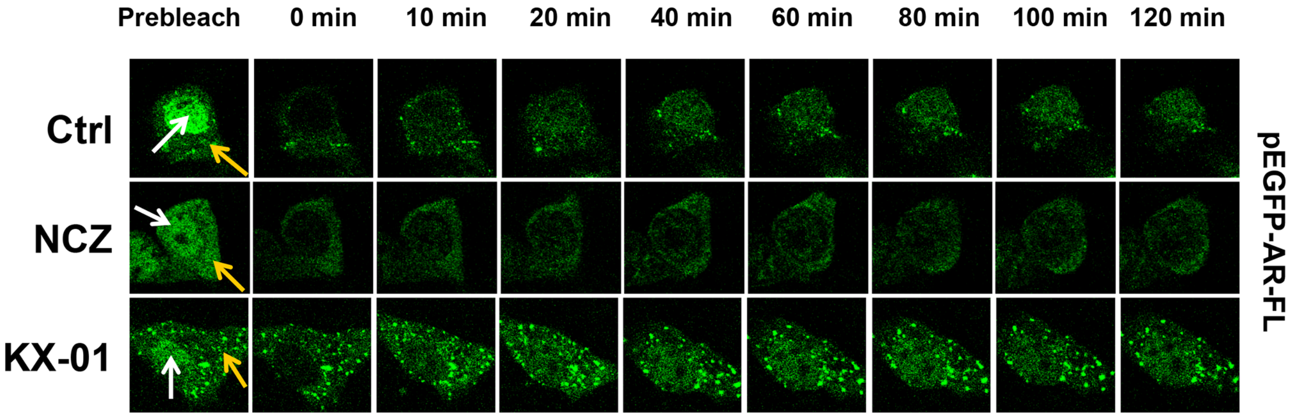

Supplementary Figure S4: FRAP images for COS-7/EGFP-AR-FL cells treated with additional microtubule inhibitors. Nocodazole (NCZ) was used at 5 μg/ml and KX-01 was at 100 nM.

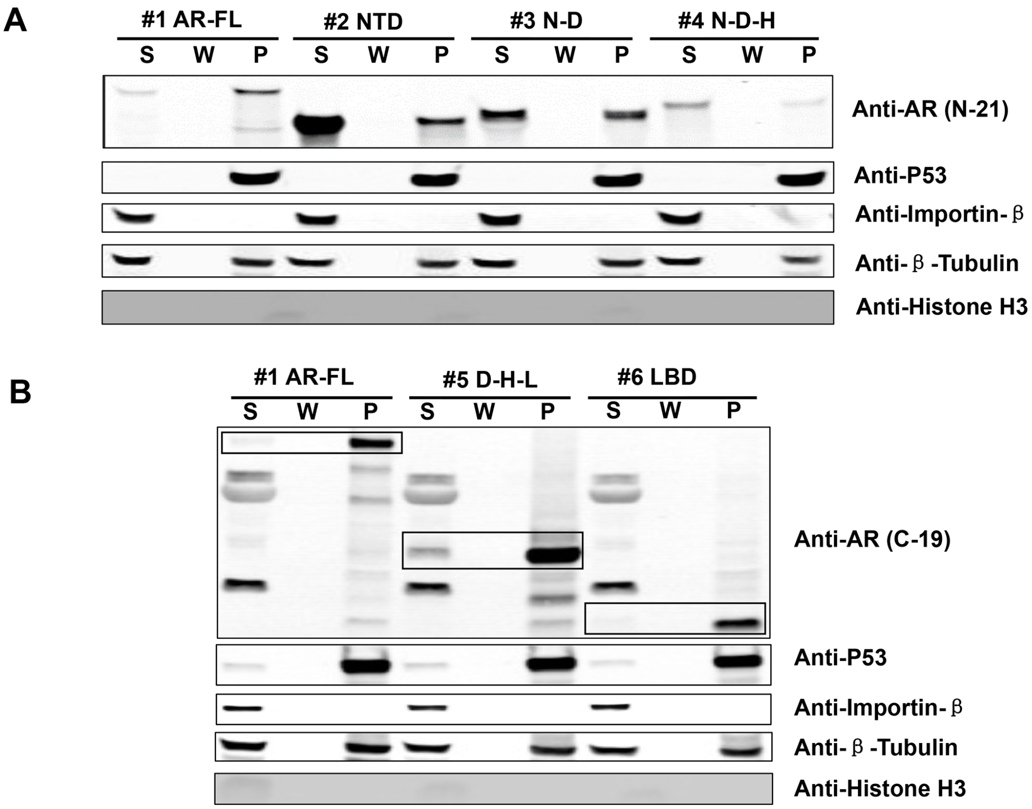

Supplementary Figure S5: MT-binding assays for AR deletion constructs. A., the C-terminal deletion constructs were probed with an antibody recognizing the N-terminus of AR (N-21). **B.**, the N-terminal deletion constructs were probed with an antibody recognizing the C-terminus (C-19). The expected bands are outlined by the rectangles.
